# Commentary on competency-based medical education and scholarship: creating an active academic culture during residency

**DOI:** 10.1007/s40037-015-0217-5

**Published:** 2015-10-08

**Authors:** Teresa M. Chan, S. Luckett-Gatopoulos, Brent Thoma

**Affiliations:** 1 Division of Emergency Medicine, 2nd Floor McMaster Wing, Hamilton General Hospital, 237 Barton St E, L8L 2X2 Hamilton, ON Canada; 2 Division of Emergency Medicine, Department of Medicine, McMaster University, Hamilton, Ontario Canada; 3 Emergency Medicine, University of Saskatchewan, Saskatoon, Canada

Successful organizations strive to understand and harness institutional culture in the pursuit of their mandates [1, 2]. In this issue, Bourgeois and colleagues observe that it is a time of great transformation in the field of medicine [3]. These authors suggest that the curricular changes required by nascent competency-based medical education (CBME) frameworks [4−6] create an opportunity to facilitate the development of an organizational culture of academic scholarship in postgraduate medical training programmes [3].

## The role of organizational structure and culture

Bourgeois and colleagues astutely acknowledge the substantive role that institutional culture plays in optimizing academic productivity [3]. Harnessing the urgency for change created during transition to CBME, however, is but one element in a complex series of change management techniques required to improve the scholarly output of postgraduate medical education programmes [1, 7, 8].

There is substantial non-medical literature on organizational culture; much of this theory is found within the leadership and management canons [2, 7−9]. Authorities within these domains suggest that innovative research and scholarship requires a culture that values these pursuits [2, 9]. Importantly, institutions that are successful in producing exceptional academic scholarship align the culture of their organizations with these goals [7, 9].

For more than a decade, medicine has characterized the academic physician and striven to understand the factors that create a rich academic environment and its converse, an environment that discourages academic pursuit [10]. In 2002, Souba wrote about the factors that contribute to an unpleasant workplace environment for academic physicians. These included heavy workloads with poor compensation, lack of appreciation and recognition, limited time for research and teaching, and failures of collegiality and teamwork. Souba also suggested a solution: engaging physicians in patient care, research, and education through personal and organizational leadership. In other words, creating an institutional culture that supports and facilitates academic engagement concrete commitment to cultural change. Bourgeois and colleagues [3] rightly point out that, despite Souba’s paper [10] being a decade old, physician and resident engagement in scholarly pursuit remains inadequate.

The shift towards CBME provides an opportunity for change within the academic medical training system, but CBME’s potential for improving scholarship among trainees will be foiled if the challenges posed by longstanding institutional culture are not addressed.

### The challenge of culture

An organization’s culture may include many barriers to change [7]. These barriers are often difficult to identify, and many institutions fail to address them appropriately. The result is a failure of well-intentioned change initiatives [1]. As CBME is implemented, educators will not identify and overcome these barriers without an intimate understanding of their institutional culture and how it drives behaviour. The following case illustrates a fictional example of a department struggling to meet its scholarly potential.

Illustrative case Dr. Williams, head of the Department of Emergency Medicine at the University Medical Centre, is faced with a challenge. His department is suffering a brain drain. Residents are completing their training and leaving the centre, enticed by non-academic, clinical jobs in community centres. Despite strong training, those who have stayed have little interest in pursuing research careers. As a result, very little scholarly work is being produced.

Dr. Williams feels that the programme and curriculum are failing. He realizes that changes must be made to instil in the residents the desire for scholarly pursuit. He recognizes the opportunity to introduce these changes with the implementation of a CBME curriculum at his institution. Dr. Williams hopes that this will result in an increase in the residents’ scholarly pursuits during training that will carry forward in their transition to faculty members.

Unfortunately, there are few scholarly mentors within the department, and also very few non-physician research associates. Several senior faculty members are engaged in research and are quite successful in obtaining grants, but they are approaching retirement and complain that they do not have time to supervise residents; thus, these larger grants are not shared with junior researchers and residents. There is a head of research, Dr. Anand, but she is very busy as the vice-chair of education and residency research coordinator.

Under Dr. Anand’s supervision, some residents have won local university-level grants earmarked for fostering resident research interest. The residency director, Dr. Tomaz, has recently noted that the department does not specifically devote any time or resources to actively fostering resident or student scholarship locally, relying on these university-level grants almost exclusively. Most residents are not interested in research, though a few express an interest in medical education. Those residents have latched onto a single mentor, Dr. Lee, who has confessed to Dr. Williams that she feels out of her depth in this supervisory role, as she has just begun her formal training in medical education.

Dr. Williams recognizes that there is a problem with the institutional culture in his department. There is little support for scholarly work, no structure in place to retain scholarly faculty, and mentorship is lacking. Scholarly successes are rarely celebrated or rewarded. As a result, the department has difficulty attracting new, academically-inclined residents and faculty members.

Dr. Williams is unsure how best to leverage the implementation of CBME to improve the scholarly output of his department.

## The four frames model by Bolman and Deal

Bolman and Deal published a popular approach to assessing organizations using a ‘Four Frames’ conceptual model based in management, leadership, and organizational theory literature [7]. This model helps define the current state of an institution and identify changes required to transform its culture into the desired state.

The ‘Four Frames’, along with some guiding questions for assessing their status, are detailed in Table 1. The structural frame refers to the organizational design of the institution, and outlines how work is divided and coordinated. The human resources frame addresses the interactions between people within the organization and how well the intrinsic needs of the individuals are met. The political frame explores relationships and power dynamics within the organization and how the organization interacts with external bodies. Finally, the symbolic frame concerns the symbols, stories, beliefs, values, and practices of the people within the organization. Each of the frames affects the development and evolution of organizational culture, but the symbolic frame includes its most tangible features [7].

**Table 1:** 

Structural	• Does the organizational structure support scholarship? (e.g. Do you have positions for a director of research? A director of education scholarship?)
	• Are key faculty members responsible for spearheading scholarship initiatives?
	• Is there appropriate administrative, financial, methodological, and statistical support in place?
	• How are the faculty members and administrative people organized and coordinated?
	• Have research competencies been identified?
	• Has a research curriculum been developed?
Human resources	• Do you have individuals with the right expertise available in your department? Are there enough of these experts? Is there a plan in place for retaining and advancing them?
	• Do they feel empowered to meet their objectives?
	• Do your people have the support (e.g. infrastructure as well as financial, methodological, and statistical support) to meet their goals?
	• Have they been provided with sufficient training and time to meet the department’s goals?
Political	• Who holds the power to affect change?
	• Who are other stakeholders?
	• Who are your supporters?
	• Who are your sceptics or critics?
	• What conflicts and coalitions exist within your organization?
	• Have relationships been developed with other internal (e.g. a research division) and external supporting bodies (e.g. research centres, granting organizations)?
Symbolic	• Is the scholarly mission of the department clear and consistent with its other priorities?
	• What symbols (e.g. items/events/people that cause department members to think of the department’s scholarship), stories (e.g. narratives describing key scholars from the department), beliefs, values, and practices are associated with scholarship?
	• How is scholarly excellence celebrated?
	• Is there alignment between your organization's values and greater social cultural phenomena?
	• Are there any competing symbolic issues to which your organization already subscribes? (e.g. Work-life balance; clinical workload)

Bolman and Deal’s ‘Four Frames’ framework, and the questions outlined above, can facilitate the examination of a department’s barriers to enhancing scholarly output. Table 2 contains the case analysis for the illustrative case we presented earlier in the paper.

**Table 2:** 

Frame	Problems	Solutions
Structural	• The organizational structure of the department was composed in an ad hoc manner based on the academic physicians who were available.	• Restructure the department to ensure that the academic workload is split equitably.
	• Single academics hold multiple positions.	• Reduce the administrative, clinical, and educational workload of researchers in the group.
	• The research funding that is available is largely utilized by senior researchers.	• Support a mentorship programme to foster relationships between residents and senior research staff.
		• Modify the system for distributing resources to ensure that junior researchers receive monetary support for their projects.
Human resources	• Residents are interested in medical education but there are few faculty trained in this area.	• Recruit faculty with expertise in medical education.
	• There are no non-physician researchers associated with the department and very few non-physician collaborators.	• Hire a non-physician research associate to support resident research and/or establish co-mentorship models with other university departments.
	• Many of the clinicians hired by the department have no interest in scholarly pursuits.	• Empower junior researchers within the department by supporting their professional development.
		• Modify hiring criteria for residents and physician staff to ensure that interests in scholarly pursuits is prioritized.
Political	• The department has few relationships with other parts of the organization.	• Develop a collaborative relationship with the institution’s research groups and other departments.
	• The department does not devote a substantive portion of its funds to support scholarly pursuit.	• Consider likely allies and opponents for the proposed changes and meet with them to define and address concerns.
	• Few faculty are interested in financially supporting scholarly pursuits.	• Meet with upper administration and request aid and resources for the development of a department that is scholarship-focussed.
	• Department leadership is supportive of the goal of increasing scholarship but has not taken concrete action to support it.	
Symbolic	• Scholarly achievement is not celebrated.	• Develop rewards and incentives that encourage participation in scholarly pursuits.
	• Some group values (e.g. family, clinical care, financial success) can be construed to contradict prioritizing scholarship.	• Bring together members of the department to develop a shared vision and mission statement.
	• The residency programme does not have a strong scholarly tradition.	• Prominently feature scholarly successes of department members in appropriate forums. Assist in disseminating research using the institution’s resources.

### ‘Culture eats strategy for lunch’

This quote, attributed to management guru Peter Drucker, succinctly encompasses the challenges faced by those who try to change the status quo. As outlined by Bourgeois [3], the implementation of CBME will require substantial change in how we educate and assess medical learners and create opportunities for encouraging a culture of scholarship. While these changes may provide an important opportunity for insightful leaders to shift the scholarly culture of their organizations, broad change initiatives are challenging. A detailed organizational analysis based on established principles from the leadership literature such as Bolman and Deal’s [7] Four Frame Model can provide valuable early insights into potential problems and sources of resistance.
